# Forensic approach in cases of anabolic-androgenic steroid abuse and cardiovascular mortality: insights from autopsy, histopathology, immunohistochemistry and toxicology

**DOI:** 10.3389/fcvm.2025.1585205

**Published:** 2025-10-09

**Authors:** Nicola Di Fazio, Gianpietro Volonnino, Michele Treglia, Giuseppe Delogu, Tommaso Bubbico, Raffaella Rinaldi, Raffaele La Russa, Aniello Maiese

**Affiliations:** ^1^ Link Campus University, Rome, Italy; ^2^ Sapienza University, Rome, Italy; ^3^ University of Tor Vergata, Rome, Italy; ^4^ University of L’Aquila, L'Aquila, Italy

**Keywords:** steroids, abuse, cardiomyopathy, sudden cardiac death, pathology, forensics, toxicology, immunohistochemistry

## Abstract

The abuse of anabolic-androgenic steroids (AAS) is associated with numerous adverse cardiovascular effects, including ventricular hypertrophy, myocardial fibrosis, and sudden cardiac death (SCD), which is a term that identifies a sudden death occurred due to cardiac conditions, congenital or acquired, particularly among young athletes and bodybuilders. This systematic review examines cases of AAS-related deaths, with a particular focus on autoptic, histopathological, immunohistochemical, and toxicological findings that highlight cardiac remodeling and myocardial damage. Numerous fatal cases were analyzed, primarily involving young men with a history of AAS abuse. Autopsy examinations revealed significant cardiac abnormalities such as left ventricular hypertrophy, coronary thrombosis, and dilated cardiomyopathy. Histopathological analyses showed focal myocardial necrosis, myocardial fiber disarray, and interstitial fibrosis, while immunohistochemical studies confirmed the presence of markers such as troponin T, fibronectin, and the C5b-9 complement complex, indicating inflammation, fibrosis, and necrosis. Toxicological analyses frequently detected testosterone, stanozolol, trenbolone, and nandrolone in blood, urine, and hair samples, confirming prolonged use of these substances. The results suggest that AAS-induced hypertrophy and fibrosis contribute to the pathogenesis of fatal arrhythmias and sudden cardiac death, even in the absence of pre-existing coronary artery disease. This review highlights the importance of integrating histopathological, immunohistochemical, and toxicological analyses with autopsy findings in forensic investigations to accurately identify AAS-related deaths and develop prevention strategies to reduce the abuse of these substances, particularly among young athletes and bodybuilders.

## 1 Introduction

Anabolic-androgenic steroids (AASs) encompass testosterone and its synthetic analogs, which exert their effects by binding the androgen receptor (AR) in the cytoplasm and subsequently translocating into the nucleus (1–4). These compounds primarily target skeletal muscle, inducing dose-dependent hypertrophy of type I and II muscle fibers and enhancing exercise tolerance through reduced fiber damage and augmented protein synthesis (1, 5, 6). In clinical settings, AASs are administered to manage conditions such as severe burns, various forms of hypogonadism, short stature, HIV wasting syndrome, and cachexia-related disorders, as well as to mitigate side effects from clinical conditions like osteoporosis and aplastic anemia (1, 7–9). Table 1 depicts the main AASs distinguishing by administration method.

**Table 1:** Main AASs distinguishing by administration method (1).

Oral agents (17α-alkyl derivates)	Injectable agents (17β-ester derivates)
Methandrostenolone	Testosterone
Methyltestosterone	Nandrolone
Oxandrolone	Boldenone
Oxymetholone	Methenolone
Stanozolol	Trenbolone
Ethylestrenol	Stanozolol
Fluoxymesterone	Dromostanolone
Danazol	

Although the molecular mechanisms have not been fully elucidated, several hypotheses have been proposed to explain how anabolic-androgenic steroids (AASs) may increase the risk of cardiovascular diseases and, consequently, sudden cardiac death. One such hypothesis involves coronary vasospasm. As previously mentioned, AAS molecules bind to androgen receptors (ARs), which are ubiquitously expressed, including in the smooth muscle cells of the arterial tunica media. This binding activates voltage-dependent L-type calcium channels, voltage-gated potassium channels, and calcium-activated potassium channels. Under physiological conditions, this cascade would typically induce vasodilation; however, at supraphysiological doses, it paradoxically leads to coronary vasospasm, clinically manifesting as precordial pain and elevated myocardial injury markers, despite the absence of electrocardiographic changes (10).

Additional studies have shown that the use of supraphysiological doses of AASs is frequently associated with cardiac arrhythmias, particularly during physical activity. These include delayed conduction of the QRS complex, ventricular fibrillation, supraventricular fibrillation, and ventricular ectopic beats. Such arrhythmic events may be linked to autonomic dysregulation, as AASs are known to interfere with several neurotransmitter systems, including the dopaminergic, serotonergic, and GABAergic pathways. Nandrolone, for instance, has been associated with an increased risk of ventricular fibrillation. Moreover, evidence from the literature indicates that supraphysiological levels of AASs induce biventricular remodeling, including alterations in the cardiac conduction tissue responsible for impulse depolarization (10).

Besides cardiovascular impairment, prolonged use of anabolic–androgenic steroids (AASs) can lead to numerous adverse effects involving organs and systems other than the cardiovascular system, which are listed below.

At the hematologic level, it is well established that administration of AASs induces erythrocytosis, namely an increase in hematocrit and/or hemoglobin levels. This phenomenon is very common in older adults receiving testosterone replacement therapy (TRT), but it may also occur in younger individuals. The underlying mechanism has not been fully elucidated, but it is hypothesized to involve changes in the regulation of erythropoietin and hemoglobin expression, along with concomitant suppression of hepcidin. In young subjects, hemoglobin levels have been shown to rise by 1.4 g/dl after 20 weeks of daily administration of 600 mg of testosterone enanthate; this translates into an ∼4% increase in hematocrit relative to baseline (11). The resulting increase in blood viscosity, as is well known, elevates the risk of venous thromboembolism (VTE) and pulmonary thromboembolism (PE) (12).

Another adverse effect of long-term AAS use described in the literature is hepatotoxicity, which occurs particularly with testosterone derivatives that have undergone 17α-alkylation at the C-17 residue (i.e., methandrostenolone, oxymetholone, danazol, epistane, fluoxymesterone, stanozolol, norethandrolone, oxandrolone, and methyltestosterone). Biochemically, this typically manifests as a mild increase in serum transaminases (AST, ALT), lactate dehydrogenase (LDH), and gamma-glutamyl transpeptidase (GGT); jaundice and pruritus are uncommon (11). Other reports suggest a correlation between prolonged use of these compounds and hepatic peliosis—an alteration of hepatic circulation in which blood flows through dilated hepatic sinusoids, accompanied by multiple focal cysts within the parenchyma (13)—and, in the most severe cases, hepatic adenoma or hepatocellular carcinoma (11, 13). The biomolecular mechanism underlying these alterations is unknown, but hyperactivation of androgen receptors (ARs) leading to the generation of reactive oxygen species (ROS) has been proposed (11).

There is also evidence of renal involvement in AAS users. Studies have documented albuminuria—i.e., the presence of albumin in the urine—in 16% of AAS users, as well as elevated serum creatinine, both indicative of kidney injury. Increased serum creatinine may also be influenced by dietary creatine intake—a common practice among bodybuilders to increase muscle mass. In any case, the mechanism of injury remains unclear (11). Proposed hypotheses include: direct glomerular toxicity of these compounds; hyperfiltration due to increased body mass index (similarly to what is observed in obesity); or glomerulosclerosis (although the latter mechanism has been demonstrated only in murine models) (13).

The hypertensive effect of AASs is also well recognized. Studies have shown that systolic and diastolic blood pressure increase by 7 and 3 mmHg, respectively, compared with baseline in AAS users. This is thought to be due to up-regulation of thromboxane A2 expression, increased synthesis of norepinephrine, the action of endothelin-1, and activation of the renin–angiotensin–aldosterone system (11).

It is also necessary to recall the effects of AASs on the endocrine system, including the reduction of endogenous testosterone production via negative feedback on the hypothalamic–pituitar*y* axis; this adversely affects spermatogenesis, as suppression of endogenous testosterone ultimately halts the process. Among the most common endocrine alterations in AAS users is gynecomastia—i.e., enlargement of the glandular component of the breast—which occurs in 7% to 19% of individuals who have completed an AAS cycle. This is essentially due to an imbalance, secondary to the use of these compounds, in the ratio of circulating androgens to estrogens, with a marked predominance of the latter. Finally, androgenetic alopecia is among the adverse effects associated with AAS use (11).

It seems that there is also an association between AASs abuse and psychopathology and, even if the correlation between these two has to be more studied, Busardò et al. published a decade ago a paper that highlighted the link between the use of Nandrolone Decanoate, which is an anabolic androgenic steroid, and an aggressive behavior (14). These compounds can be administrated alone or combined, between them, or, for example, with other kind of PEDs, such as the GHB (15).

**Figure 1:**
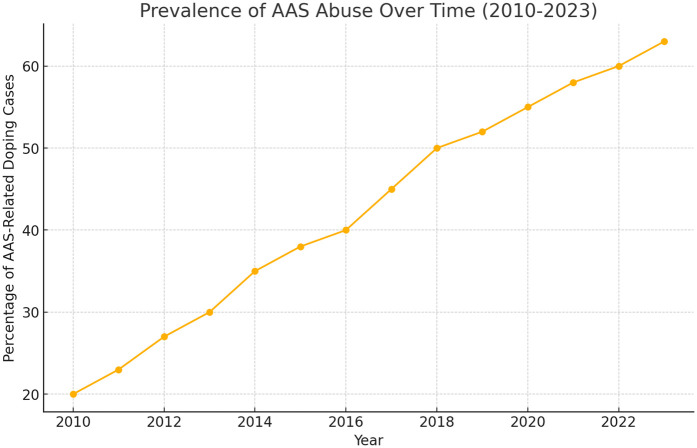
Prevalence of AAS abuse over time worldwide.

Despite their therapeutic applications, AASs account for 50% of doping cases worldwide; their misuse is also increasing among non-athletes seeking aesthetic benefits (16–18) (Figure 1). Adverse cardiovascular effects include lower plasma HDL levels (16, 19) and sudden cardiac death, often linked to hypertrophic cardiomyopathy, especially in young athletes (16, 20–23). Other organ systems - reproductive, musculoskeletal, endocrine, renal, and hematological—can also be affected, and users may experience psychological disturbances such as mood instability and aggressive behaviour (14, 16). In the Italian jurisdiction, in order to combat the use of doping substances in sport activities, a law titled “Regulation of health standards in sport activities and the fight against doping” has been enacted (Law of December 14, 2000, n.376) (24).

This review addresses AAS-related fatalities, focusing on cardiovascular pathologies, autopsy findings, and histopathological and immunohistochemical evidence. It aims to elucidate the underlying pathophysiological mechanisms of AAS-induced cardiac damage, which remain only partially understood.

## 2 Materials and methods

The present systematic review was prepared according to the Preferred Reporting Items for Systematic Review (PRISMA) standards (25).

First, the P.I.C.O. the method used to formulate research questions clearly and systematically, especially in the medical and healthcare fields, facilitating the conduct of systematic reviews and clinical studies, was developed.

### 2.1 Inclusion criteria

We selected the study to be included in the present systematic review based on the following characteristics:

P (Population):

• Deceased individuals with a history of anabolic-androgenic steroid (AAS) use.
• Bodybuilders, athletes, or individuals who used AAS for aesthetic purposes or to enhance physical performance.

I (Intervention):

• Autopsy and histopathological examination of the hearts of AAS users.
• Immunohistochemical analyses to detect cellular damage or specific biomarkers (e.g.: troponin, fibronectin, C5b-9).

C (Comparison):

• Deceased individuals who did not use AAS (control group).
• Macroscopic and microscopic differences between the hearts of AAS users and non-users.

O (Outcome):

• Evidence of cardiac abnormalities (ventricular hypertrophy, myocardial fibrosis, myocyte necrosis, early atherosclerosis).
• Frequency of arrhythmias, myocardial infarction, dilated or hypertrophic cardiomyopathy in AAS users.
• Immunohistochemical biomarkers related to myocardial damage.

Research Question:

What are the macroscopic and microscopic effects of AAS on the heart, detectable through autopsy, histopathology, and immunohistochemistry?
Is there a correlation between AAS use and the occurrence of cardiac abnormalities detectable post-mortem?

### 2.2 Exclusion criteria

The exclusion criteria were established as follows:

1. Studies on populations with pre-existing comorbidities
2. Studies on subjects with congenital heart disease not related to AAS use
3. Studies lacking autoptic, histopathological, or immunohistochemical data
4. Studies addressing clinical outcomes without autopsy confirmation
5. Studies involving subjects who did not die from cardiovascular conditions
6. Letters to the editor/book's chapters.

### 2.3 Information source and search process

A systematic literature search and a critical appraisal of the collected studies were conducted. A bibliographic search using 3 databases (PubMed, Embase, Web of Science) has been carried out from the inception of these databases to 15 March 2025 by entering the following keywords:

• “Anabolic Androgenic Steroids” AND “Autopsy”;
• “Anabolic Androgenic Steroids” AND “Sudden Cardiac Death” AND “Histopathological Findings”;
• “Sudden Cardiac Death” AND “Steroids” AND “Anabolic” AND (“Histology” OR “Immunohistochemistry”);
• “Sudden Cardiac Death” AND (“Methandrostenolone” OR “Methyltestosterone” OR “Oxandrolone” OR “Oxymetholone” OR “Stanozolol” OR “Ethylstrenol” OR “Fluoxymestrone” OR “Danazol”) AND (“Histology” OR “Immunohistochemistry”);
• “Sudden Cardiac Death” AND (“Testosterone” OR “Nandrolone” OR “Boldenone” OR “Methenolone” OR “Trenbolone” OR “Stanozolol”) AND (“Histology” OR “Immunohistochemistry”).

The articles of interest were analyzed in full-text version and selected by two initial reviewers (T.B., G.V.); the results were compared with those of two other reviewers (N.D.F., M.T.), following an open discussion among all authors. Articles that respected the inclusion criteria were admitted. Only papers or abstracts in English were included in the search. No unpublished or grey literature was searched.

Then, the articles, in order to write the following review, were selected from PubMed and Scopus.

## 3 Results

### 3.1 Study selection

**Figure 2:**
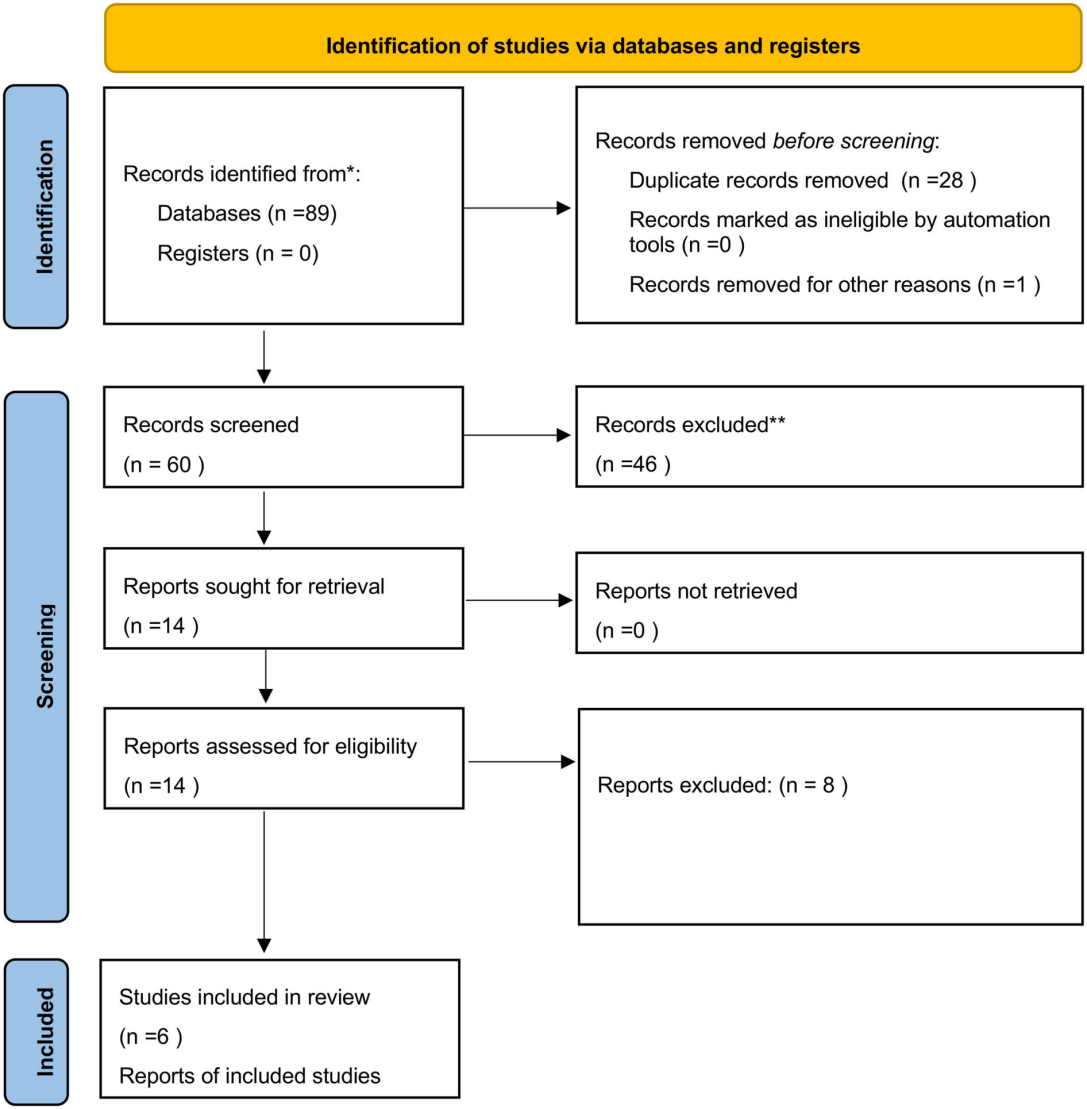
PRISMA 2020 flow diagram adopted for the present review (27).

A total of 89 articles were identified; 28 were excluded as duplicates, and one was excluded because it was not accessible. The titles and abstracts of the remaining 60 articles were then reviewed; 46 were discarded as they were not relevant to the research topic (as defined by the P.I.C.O. framework). Of the 14 remaining articles, 6 were included in the review, while 8 were excluded based on the exclusion criteria outlined in the previous section. Additionally, 2 articles from other sources were added to the 6 selected articles (Figure 2).

### 3.2 Study characteristics

Cecchi et al. (26) conducted a study analyzing autoptic, histopathological, immunohistochemical, and toxicological elements in order to examine cardiac remodeling associated with AASs abuse. The study focused on two cases involving young male bodybuilders, aged 20 and 23, who both died suddenly and had a history of AASs abuse, particularly metenolone and nandrolone, to determine whether there was any underlying cardiac pathology. The autopsy revealed significant cardiac abnormalities in both cases. Cardiomegaly was observed, with heart weights of 440 g and 430 g, respectively, along with ventricular hypertrophy. The thickness of the interventricular septum ranged between 21 and 22 mm, while the free wall of the left ventricle measured between 19- and 20-mm. Ventricular dilation was also present. Despite these morphological abnormalities, no evidence of coronary atherosclerosis or thrombosis was found. This led to the conclusion that AAS abuse directly contributed to the observed pathological changes, which are typically associated with traditional ischemic heart disease. Histopathological examination revealed significant cardiac remodeling. There was widespread interstitial and perivascular fibrosis, subendocardial fibrosis, and areas of fibrofatty replacement. Additionally, prominent disarray of myocardial fibers, focal necrosis, and colliquative myocytolysis were observed. These findings suggested chronic myocardial damage resulting from prolonged exposure to AASs. The immunohistochemical analysis provided deeper insights into the mechanisms of myocardial injury. CD68-positive macrophages were diffusely distributed throughout the myocardium, with a predominance of CD163-positive M2 macrophages, suggesting a reparative rather than inflammatory response. Hypoxia-inducible factor 1-alpha (HIF-1α) was detected in endothelial cells, particularly in fibrotic areas, indicating a chronic hypoxic stress mechanism. Apoptotic activity was observed and confirmed by widespread TUNEL positivity in both myocardial and endothelial cells. Caspase-3 staining further highlighted apoptotic changes in cardiomyocytes and endothelial cells. Caspase-3, defined as an effector caspase, is the terminal effector of both mitochondria-dependent (intrinsic) and mitochondria-independent (extrinsic) apoptosis. Many studies have been conducted on the importance of mitochondria in both apoptosis and necrosis; both phenomena, in fact, are triggered by the release of apoptogenic proteases or activating proteases from these cellular organelles (27). The mix between training and AASs use could lead to destruction of mitochondria and to the release of caspases until the apoptotic death of the cell (28–30). Toxicological analysis conducted on the two deceased bodybuilders revealed the presence of androgenic-anabolic steroids (AAS) in their systems. In the first case, a 20-year-old bodybuilder was found to have 5 ng/ml of nandrolone and 7 ng/ml of metelonone in his urine. Similarly, in the second case, a 23-year-old bodybuilder tested positive for 7 ng/ml of nandrolone. These results, obtained through Gas Chromatography-Mass Spectrometry/Mass Spectrometry (GC-MS/MS), confirmed prolonged steroid use in both individuals prior to their deaths **(**26). Ultimately there are two studies—published respectively by Satoh et al. and Ostrander et al.—which remarked that the genetics may have an impact on some reactions by mitochondria if they are exposed to AASs and hard training (31, 32).

Favretto and colleagues (33) presented a forensic investigation regarding a twenty-four-year-old male bodybuilder who died suddenly; the cause of death was attributed to abuse of AASs and clenbuterol. The man was found unconscious in a hotel room the day before a bodybuilding competition he was supposed to participate in. During the on-site inspection, numerous drugs were found, including clenbuterol, fluoxymesterone, torasemide, furosemide, and levothyroxine. The autopsy revealed significant cardiac abnormalities, including notable cardiomegaly; at the level of the coronary arteries, there was hyperplasia of the intimal layer, which was not hemodynamically significant. Multiorgan congestion was observed in the lungs, liver, kidneys, adrenal glands, spleen, and brain; the lungs, in particular, were severely edematous, and hemorrhagic extravasation was present. The meningeal veins were also congested. Histopathological analyses confirmed diffuse multiorgan congestion and pulmonary edema but did not reveal the presence of thrombi or ischemic necrosis in the cardiac structures. No underlying cardiac pathology was identified in this case either. Immunohistochemical investigation was not performed. Toxicological tests detected the presence of clenbuterol in both blood and urine (1 ng/ml). Additionally, a metabolite of drostanolone (2-alpha-methyl-androsterone) was found in the urine (202 ng/ml); the testosterone/epitestosterone (T/E) ratio measured in the urine was 11 (normal value ≤4). Hair analysis provided further information, demonstrating prolonged and improper use of substances; clenbuterol (25 pg/mg), stanozolol (42 pg/ml), trenbolone, drostanolone, metenolone, methandienone, and numerous testosterone esters (cypionate, decanoate, propionate, undecanoate) were identified. Although packages of diuretic drugs were present at the scene, none were detected in the biological samples. Post-mortem investigations determined that the main cause of death was attributable to clenbuterol toxicity, which caused cardiac, hepatic, and metabolic complications. The absence of other pathological conditions reinforced this conclusion (33).

Lichtenfeld et al. (34) described the case of a cardiac arrest in a 13-year-old male athlete, suspected to be linked to improper use of AASs. The adolescent collapsed during a wind sprint competition at the sports field. Paramedics at the scene documented ventricular fibrillation and, although resuscitation efforts were successful, the patient suffered severe neurological damage and subsequently passed away. The autopsy revealed severe cardiomegaly; the heart weighed 465 g, far exceeding the expected 175 g. There was evidence of marked ventricular hypertrophy characterized by thickening of the free ventricular wall and septum; the left ventricular cavity had a smaller diameter than normal. Additionally, an increased amount of epicardial fat covered the heart; however, the coronary arteries were normal, with no evidence of obstruction. Beyond the cardiac abnormalities, other anomalies were found throughout the body, including adrenal atrophy with lipid depletion, fat infiltration in the liver, and signs of early development of secondary sexual characteristics such as advanced muscular development, mature genitalia, and striae on the biceps and thighs. Histological examination showed myofibril disarray, fibroblast proliferation indicative of early fibrosis, and expansion of myofibers with nuclear pleomorphism (“box-car” nuclei). There was no evidence of myocarditis or right ventricular dysplasia. Immunohistochemical examinations were not performed. The primary cause of death was attributed to early cardiac arrest following ventricular fibrillation caused by hypertrophic cardiomyopathy due to AAS abuse. The toxicological analysis in this case report was limited by the unavailability of urine samples at the time anabolic steroid use was suspected. As a result, chromatographic analysis for steroid metabolites could not be performed to confirm the presence of anabolic steroids (34).

Lusetti and colleagues (35) presented a retrospective analysis of six sudden and unexpected deaths involving male AAS users. The study highlighted pathological changes in the heart observed during forensic investigations, focusing on the role of steroid-induced cardiac remodeling in fatal outcomes. Autopsy findings frequently revealed the presence of left ventricular hypertrophy, accompanied by asymmetric thickening of the left ventricular free wall (up to 20 mm) and the interventricular septum (up to 16 mm). Heart weights were significantly increased; in one case, the heart weighed 490 g (0.54% of total body mass). Despite these findings, there was no evidence of acute myocardial infarction or significant narrowing of the coronary artery lumen. Supporting the hypothesis of AAS abuse, testicular atrophy was a recurring finding in all analyzed cases. Histopathological analyses demonstrated widespread myocardial fibrosis, with interstitial and perivascular patterns. In some cases, fibroadipose metaplasia was observed, accompanied by contraction band necrosis and segmentation of cardiomyocytes. Enlargement of intercalated discs was also noted, indicating structural cardiac remodeling often associated with arrhythmogenic potential. Immunohistochemical examinations detected occasional single cardiomyocyte necrosis, as indicated by fibronectin staining, but there was no evidence of diffuse or grouped necrosis. Analysis of the complement Csb-9 complex did not show necrotic patterns, suggesting that myocardial damage may have developed progressively (rather than acutely, as in ischemic events). Toxicological analyses confirmed the presence of nandrolone and testosterone in urine and hair samples, with an elevated T/E ratio (in one case, the ratio was 21). This further evidenced chronic AAS use, even in the absence of acute intoxication or metabolic disturbances. In each analyzed case, the cause of death was attributed to cardiac arrhythmia, inferred by excluding other potential causes through comprehensive autopsy, histological, immunohistochemical, and toxicological analyses. The findings suggested that AAS-induced cardiac hypertrophy and fibrosis likely predispose these individuals to fatal arrhythmias **(**35).

In another study by Lusetti et al. (36), five cases of sudden death involving male AAS users were analyzed, focusing on cardiac changes and systemic effects related to AAS abuse. The subjects, aged between 29 and 39 years (average age 33), used AASs for performance or aesthetic purposes. All exhibited significant muscular development, indicative of prolonged AAS use. Autopsy examinations consistently revealed cardiac hypertrophy, with left ventricular hypertrophy present in three cases and biventricular hypertrophy in one case. Heart weights ranged from 380 g to 480 g, exceeding normal values for individuals with comparable body mass. The coronary arteries showed thickening of the intimal and medial layers, with fatty streaks observed in one case. Testicular atrophy was a recurring finding in all subjects, supporting the link to steroid use. Histopathological analyses identified interstitial fibrosis and cardiomyocyte necrosis in multiple cases, with fibrotic changes predominantly affecting the left ventricle. Notably, there was no sign of acute myocardial infarction, suggesting that cardiac remodeling was a chronic process likely driven by prolonged AAS exposure. Toxicological analyses confirmed the presence of nandrolone, testosterone, boldenone, metenolone, oxandrolone, stanozolol, and clomiphene in biological samples. In several cases, additional substances such as methadone, THC, cocaine, morphine, citalopram, clozapine, venlafaxine, phenobarbital, lorazepam, and ethanol were detected, highlighting the prevalence of polypharmaceutical abuse in this population. The cause of death in all cases was attributed to cardiac arrhythmias and myocardial fibrosis, driven by AAS-induced cardiac remodeling. The presence of psychoactive substances likely exacerbated the risk of fatal arrhythmias, contributing to the sudden and unexpected nature of these deaths (36)**.**

Hernández-Guerra et al. (7) present a case of sudden cardiac death in a 24-year-old male bodybuilder with a history of anabolic-androgenic steroid (AAS) use. The individual experienced cardiorespiratory arrest at home following a New Year's celebration. Despite having no family history of dyslipidemia or cardiovascular disease, the patient had reported chest pain several months prior. Autopsy findings revealed significant cardiac pathology, including cardiomegaly (420 g), exceeding the expected heart weight. The left ventricular free wall and interventricular septum both measured 15 mm in thickness, while the right ventricular free wall was 5 mm. Severe coronary atherosclerosis was identified, with over 75% stenosis in major arteries. Acute occlusive thrombosis was observed in the left main trunk and left anterior descending (LAD) artery. Additionally, scarring was present at the intersection of the posterior wall and septum, indicative of prior ischemic events. Histopathological analysis confirmed acute myocardial infarction affecting the anterior septum and the anterior wall of the left ventricle, with evidence of subacute infarction in the apical septum and posterior left ventricular wall. There was myocyte hypertrophy, fibrosis, and small intramyocardial vessel disease, suggesting long-term cardiovascular stress and chronic ischemic damage. Toxicological analysis identified elevated concentrations of stanozolol (11.31 mg/L) and nandrolone (2.05 mg/L) in the blood, with ethanol detected at 0.90 g/L. Testosterone levels were below the quantification threshold, and no traces of tamoxifen or mesterolone were found. The cause of death was attributed to acute myocardial infarction and severe coronary atherosclerosis, directly linked to AAS consumption. This case underscores the cardiovascular risks associated with AAS abuse, highlighting the role of myocardial hypertrophy, atherosclerosis, and thrombosis in sudden cardiac events (7).

Frati et al. (1), in their systematic review, analyzed 19 fatal cases related to AAS abuse and focused on autopsy, histopathological, and toxicological findings. The cases covered in the study spanned from 1990 to 2012; they primarily involved young male athletes or bodybuilders (average age 28). Autopsy examinations revealed significant cardiac abnormalities. Left ventricular hypertrophy was observed in 14 out of 19 cases, often accompanied by myocardial fibrosis and contraction band necrosis. In more than one instance, coronary thrombi were detected, and cases of dilated cardiomyopathy were also documented. Additionally, findings such as hepatosplenomegaly, renal hypertrophy, and cerebral edema were reported; in one case, pulmonary embolism, which is the most dangerous clinical manifestation of venous thromboembolism (39), was also noted. Histopathological analyses confirmed widespread cardiac remodeling. The most common findings included focal myocardial fibrosis, cardiomyocyte hypertrophy, fiber disarray, and lymphocytic infiltration. Nuclear polymorphism and endocardial thickening were also recorded; these findings are indicative of chronic cardiac stress and maladaptive changes in the myocardium. Toxicological investigations confirmed the presence of AASs in urine, blood, and hair in 12 of the 19 cases analyzed. The most frequently detected substances were nandrolone, testosterone, stanozolol, and methandienone. Elevated testosterone/epitestosterone (T/E) ratios supported the diagnosis of steroid abuse. In the remaining cases, despite the absence of detectable AASs in biological samples, evidence from the circumstances, including testimonies and findings at the scene, indicated prolonged AAS use. The primary cause of death in most cases was sudden cardiac death (SCD) due to cardiac hypertrophy and fibrosis (1).

Esposito et al. (16) conducted a systematic review analyzing forensic and post-mortem findings in cases of death involving a history of anabolic-androgenic steroid (AAS) abuse. The study examined 137 fatal cases involving young adult males, primarily bodybuilders, athletes, and recreational users who experienced sudden cardiac death during physical activity. Autopsy findings revealed significant cardiac abnormalities, including concentric cardiac hypertrophy, left ventricular hypertrophy (LVH), coronary thrombosis, dilated cardiomyopathy, and fibrosis, particularly affecting the subepicardial and interstitial regions. Histopathological analysis demonstrated focal cardiomyocyte necrosis, hypertrophy, myofibrillar loss, edema, and contraction band necrosis. Vascular congestion and eosinophilic changes in cardiomyocytes were recurrent findings, confirming myocardial damage at the microscopic level. Immunohistochemical studies identified markers such as Troponin T, indicating myocardial inflammation, fibrosis, and necrosis. Fibronectin confirmed the presence of interstitial fibrosis, while myoglobin staining revealed fiber degeneration. The detection of the C5b-9 complement complex further supported evidence of myocardial injury and fibrosis. Toxicological analysis was performed on blood, urine, hair, gastric contents, and cerebrospinal fluid. The presence of testosterone, stanozolol, trenbolone, nandrolone, oxymetholone, and tamoxifen was frequently detected. Gas chromatography-mass spectrometry (GC-MS) and high-performance liquid chromatography (HPLC) were used to confirm the presence of these substances across multiple biological samples. The primary causes of death in these cases included ventricular fibrillation, myocardial fibrosis, arrhythmias, and coronary thrombosis, often linked to severe left ventricular hypertrophy and/or dilated cardiomyopathy (17). Table 2 was included in the review by Esposito et al. and has been reproduced here in its entirety without any modifications or reworking (16).

**Table 2:** Previous results from a systematic review conduced by Esposito et al. (16).

Reference	Study design	Number of cases	Autopsy/histopathological findings	Immunohistochemical findings	AASs/toxicological analysis samples	Toxicological method	Cause of death
Lehmann S.	Case report	1 young adult	Hypertrophy of the heart with enlargement of the ventricular walls as well as a small vascular lumen (3 mm diameter) of the right coronary artery	Immunohistochemistry staining was conducted with antibodies against primary antibodies against troponin T that showed inﬂammation, ﬁbrosis or necrosis	Testosterone, oxymetholone, stanozolol, trenbolone—Blood, urine, cerebrospinal ﬂuid, and stomach content	HPLC	Pathological changes of the heart (left ventricular hypertrophy) and atherosclerosis of the coronaries
Bertozzi G.	Case report	1 young adult	Left thigh necrotizing myofascitis	Immunohistochemistry with anti-myoglobin antibodies showed myoﬁbrillar rexis on heart samples	Testosterone, propionate, clenbuterol, stanozolol, trenbolone, oxandrolone, tamoxifen—Hair and blood	GC–MS	AASs adversely inﬂuenced the immune response, affecting leucocyte growth or activity, and antibody and cytokine production
Lichtenfeld J.	Case report	1 young adult	Left ventricular myocardium ﬁndings: foci of myoﬁbrillar disarray, proliferation of ﬁbroblasts consistent with early ﬁbrosis, and enlarged myoﬁbers with heterogeneity of nuclear size including box car nuclei	Not available	Standard urine toxicology tests were negative	Not available	Cardiac arrest attributed to hypertrophic cardiomyopathy from anabolic steroid use, with documented ventricular ﬁbrillation as the initiating arrhythmia
Fineschi V.	Case series	2 young adults	Hepatic injury, including cholestasis, peliosis hepatis, hyperplasia, ventricular ﬁbrillation	Not available	Testosterone, nandrolone, norandrosterone, etiocholanolone, noretiocholanolone, norepiandrosterone, stanozolol, hydroxy-stanozolol, epitestosterone—Urine	GC–MS	Overstimulation of the sympathetic system followed by a transient functional and structural destabilization of the sympathetic axon terminals
Fanton L.	Original article (retrospective study)	6 of 2,250 young adults	Coronary thrombosis associated with left ventricle hypertrophy, toxic, adrenergic myocarditis, dilated cardiomyopathy associated with a recent coronary thrombosis	Immunohistochemical staining was conducted with hematoxylin–phloxin–saffron (HPS) antibodies, which showed interstitial inﬂammatory cells, interstitial reticular ﬁbrosis, concentric stenosing and intimal sclerosis of the heart	- Norethandrolon - Blood	GC–MS	Various cardiac lesions: misshapen cell nuclei, myolysis, ﬁbrosis and interstitial lesions
Lusetti M.	Original article (retrospective study)	6 of 98 young adults	Pathological changes consisted of various degrees of interstitial and perivascular ﬁbrosis as well as ﬁbroadipose metaplasia and perineural ﬁbrosis within the myocardium of the left ventricle	Immunohistochemistry, antibodies against ﬁbronectin and c5b9 showed a myocardium ﬁbrosis	Testosterone, nandrolone, epitestosterone - Cardiac blood, vitreous humor, urine, cerebrospinal and pericardial ﬂuids as well as gastric contents, hair and samples of certain tissues (liver, brain, and skeletal muscle)	GC–MS	Left ventricular hypertrophy and myocardial ﬁbrosis can create a predisposition to sudden cardiac death
Montisci M.	Case report	4 young adults	Concentric cardiac hypertrophy with focal ﬁbrosis (one case), dilated cardiomyopathy with patchy myocyte death (two cases) and eosinophilic myocarditis (one case). The most typical cardiac abnormality in AAS abusers is left ventricular hypertrophy, associated with ﬁbrosis and myocytolysis	Immunohistochemical analysis through primary antibody against Troponin T showed myocytolysis in the sub-endocardial trabeculae, hypertrophic myocytes with dysmetric and dysmorphic nuclei	Testosterone, stanazolol, nortestosterone, epitestosterone - Hair and urine	GC–MS	Three cases of sudden cardiac death (SCD) and one of death due to congestive heart failure of a previously healthy athlete
Inoue H.	Case report	1 young adult	Concentric cardiac hypertrophy was macroscopically observed. In the left and right coronary arteries, atherosclerosis was generally observed within the endothelium	Immunohistochemical analysis through primary antibody against Troponin T showed myocytolysis in the sub-endocardial trabeculae, hyper-trophic myocytes with dysmetric and dysmorphic nuclei	Testosterone - Blood	Not available	Ischemic heart disease due to coronary stenosis
Far M.	Original article (retrospective study)	87 of 260 adults (1989–2009)	A signiﬁcantly greater cardiac mass among deceased users of AASs compared to individuals with no suspected use of AASs. An elevated risk of developing concentric LVH among AAS users	Not available	Testosterone, propionate, clenbuterol, stanozolol, trenbolone, oxandrolone, tamoxifen - Urine	GC–MS	Cardiac hypertrophy with a direct cardiotropic effect
Darke S.	Original article (retrospective study)	24 adults (1995–2012)	In 23 of 24 cases, substances other than steroids were detected, most commonly psychostimulants (66.7%); in nearly half, testicular atrophy was noted, as was testicular ﬁbrosis and arrested spermatogenesis; left ventricular hypertrophy was noted in 30.4%, and moderate to severe narrowing of the coronary arteries in 26.1%	Not available	Testosterone, epitestosterone - Blood, urine	HPLC	Particularly notable extensive cardiovascular disease
Thiblin I.	Case report	1 young adult	Both the foci of replacement ﬁbrosis and the perivascular inﬂammatory changes were rather moderate, and probably not severe enough to cause arrhythmia by themselves, both ﬁbrosis and myocardial inﬂammation are known risk factors for arrhythmia	Not available	Testosterone, OH-stanozolol, 16b-OH-stanozolol, boldenon - Blood, urine	GC–MS	Sudden cardiac arrhythmia possibly related to a combination of AASs and ephedrine
Hernandez-Guerra, A.I.	Case report and literature review	1 young adult	Cardiomegaly (420 g) with a ventricular thickness that was within the upper normal ranges (left ventricular free wall 15 mm, ventricular septum 15 mm, right ventricular free wall 5 mm); acute myocardial infarction at the anterior third of the septum and the left ventricle (LV) anterior wall, subacute myocardial infarction at apical septum and apical posterior LV wall	Immunohistochemical analysis with primary antibodies against troponin T showed small intramyocardial vessels disease with media hypertrophy	Stanozolol, testosterone, tamoxifen, mesterolone, nandrolone - Blood, humor	HS–GC–FID	Myocardial infarction with severe coronary atherosclerosis and acute occlusive thrombosis affecting left main trunk and left anterior descending coronary artery (LAD) (single vessel disease)
Dufayet L.	Case report	1 young adult	Yellow discoloration of the skin, nonspeciﬁc signs of asphyxia (cyanosis, pulmonary edema and congestion); Heart: occasional foci of vascular congestion in the connective tissue surrounding coronary arteries; Lungs: edematous and congestive, with some areas of alveolar hemorrhage; Mild congestion was alsoobserved in the centrilobular region of the liver as well as in both kidneys.	Immunohistochemical analysis through primary antibody against Troponin T revealed occasional foci of vascular congestion in the connective tissue surrounding coronary arteries	2,4-dinitrophenol (DNP) clenbuterol - Blood - Urine - Gastric content	GC–MSHPLC	Toxicological analysis showed high levels of clenbuterol and DNP, conﬁrming an intoxication
Dickerman R.D:	Case report	1 young adult	The heart weighed 250 g with signs of concentric hypertrophy of the left ventricle, atherosclerosis of the vessels	Not available	Testosterone, nandrolone	Not available	Cardiac hypertrophy with a direct cardiotropic effect

Reproduction of the table for courtesy of Prof. M. Esposito, Kore University of Enna, Italy.

### 3.3 Risk of bias

Despite of the breadth of the studies involved and the scientific weight of their conclusions, an unavoidable risk of bias lies on several factors affecting our analyzed evidence. First, a small but significant time interval of 7 years lies between selected papers, which implies a particular challenge in data interpretation due to the rapidly evolving research methods on the specific field. Moreover, authors affiliation comes from only two continents (Europe, America), but it must be considered that evidences caught from Esposito et al. (16) embrace also Asiatic (Japan) and Oceanic (Australia) continents. An overview of the main heterogeneities of the studies examined can be found in Table 3.

**Table 3:** Main carachteristics of the studies involved in the present review for bias risk assessment.

Heterogeneities	Lusetti et al. (35)	Frati et al. (1)	Lichtenfeld et al. (34)	Cecchi et al. (26)	Lusetti et al. (36)	Hernandez Guerra et al. (7)	Esposito et al. (16)	Favretto et al. (33)
Year	2015	2015	2016	2017	2018	2019	2021	2022
Country	Italy	Italy (but cases from other countries are reported)	USA	Italy	Italy	Spain	Italy	Italy
Type of study	Retrospective analysis	Systematic Review	Case Report	Case Series	Setrospective case series	Case Report	Systematic Review	Case Report
Number of cases	1	19	1	2	6	1	137	1
Sex	M	M (17/19 cases); F (2/19 cases)	M	M	M	M	M	M
Range of age	39	18–37	13	20–23	29–39	24	Youth (not specified age)	24
Autopsy	Yes	Yes	Yes	Yes	Yes	Yes	Yes	Yes
Histological examination	Yes	Yes (but results are reported about 17/19 cases)	Yes	Yes	Yes	Yes	Yes (but results are reported about 136/137 cases	Yes
Immunohistochemistry examination	Yes	No specific findings in 1/19 cases; not specified in 18/19 cases	No specific findings	Yes	No specific findings	No specific findings	Yes (but results are reported about 21/137 cases	Yes
Toxicological examination	Yes	Yes (results reported in 12/19 cases)	Performed but not available	Yes	Yes	Yes	Yes	Yes

## 4 Discussion

Table 4 (46–48) was compiled by incorporating data from individual case reports as well as those presented in the review by Frati et al. (1). The reported data included:

• Name of the study;
• Number of reported cases;
• Age;
• Sex;
• Height (cm);
• Weight (kg);
• Body mass index (BMI);
• Hearth weight (g);
• LV Wall thickness (mm);
• RV Wall Thickness (mm);
• IS Wall thickness (mm);
• Other autoptic findings;
• Histological findings;
• Immunohistochemical findings.

**Table 4:** Data incorporation of AAS deaths from individual case reports as well as those presented in the review by frati et al. (1).

Study	Number of cases	Age	Sex	Height (cm)	Weight (kg)	BMI	Hearth weight (g)
Cecchi et al. 2017 (26)	1	20	M	190	87	28.41	440
Cecchi et al. 2017 (26)	2	23	M	175	106	34.61	430
Favretto et al. 2022 (33)	1	24	M	–	–	–	–
Lichtenfeld 2016 (34)	1	13	M	–	–	–	465
Hernandez-Guerra et al. 2019 (7)	1	24	M	178	85	26.8	420
Lusetti et al. 2015 (35)	1	39	M	178	90	28.4	490
Lusetti et al. 2018 (36)	1	32	M	–	–	–	390
Lusetti et al. 2018 (36)	2	32	M	–	–	–	360
Lusetti et al. 2018 (36)	3	33	M	–	–	–	425
Lusetti et al. 2018 (36)	4	39	M	–	–	–	480
Lusetti et al. 2018 (36)	5	29	M	–	–	–	340
Ferenchick et al. 1991 (37) (by Frati et al. (1)	2	22	M	–	–	–	–
Luke et al. 1990 (38) (by Frati et al. (1)	1	27	M	–	96.6	–	530
Kennedy et al. 1993 (39) (by Frati et al. (1)	3	18	M	–	–	–	410
Kennedy et al. 1993 (39) (by Frati et al. (1)	4	24	M	–	–	–	440
Dickerman et al. 1996 (40) (by Frati et al. (1)	5	26	M	182	136	41.1	440
Hausmann et al. 1998 (41) (by Frati et al. (1)	6	23	M	-–	94	–	500
Fineschi et al. 2001 (42) (by Frati et al. (1)	7	32	M	189	90	25.2	450
Fineschi et al. 2001 (42) (by Frati et al. (1)	8	29	M	166	72	26.1	390
Di Paolo et al. 2007 (43) (by Frati et al. (1)	9	29	M	190	127	35.2	490
Di Paolo et al. 2007 (43) (by Frati et al. (1)	10	27	M	190	100	27.7	360
Di Paolo et al. 2007 (43) (by Frati et al. (1)	11	37	F	162	71	27.1	310
Di Paolo et al. 2007 (43) (by Frati et al. (1)	12	31	M	175	79	25.8	400
Fineschi et al. 2007 (44) (by Frati et al. (1)	13	29	M	166	72	26.1	380
Fineschi et al. 2007 (44) (by Frati et al. (1)	14	30	M	178	90	28.4	400
Thiblin et al. 2009 (45) (by Frati et al. (1)	15	29	F	172	76	25.7	331
Montisci et al. 2012 (46) (by Frati et al. (1)	16	32	M	180	110	33.9	450
Montisci et al. 2012 (46) (by Frati et al. (1)	17	31	M	172	120	40.6	900
Montisci et al. 2012 (46) (by Frati et al. (1)	18	32	M	178	94	29.7	580
Montisci et al. 2012 (46) (by Frati et al. (1)	19	25	M	185	125	36.5	390
Study	LV WT (mm)	RV WT (mm)	IS WT (mm)	Other autoptic findings	Histological findings		Immunohistochemical findings
Cecchi et al. 2017 (26)	19	5	21	Cardiomegaly, biventricular dilatation, normal coronary arteries, liver congestion, lung emphysema	Hypertrophy, interstitial and perivascular fibrosis, hyaline degeneration		CD68 + macrophages, TUNEL + cells, caspase 3 + cells
Cecchi et al. 2017 (26)	20	7	22	Cardiomegaly, concentric LV hypertrophy, normal coronary arteries, liver and lung congestion	Hypertrophied myocytes, interstitial and perivascular fibrosis, minimal myocardial necrosis		CD68 + macrophages, TUNEL + cells, caspase 3 + cells
Favretto et al. 2022 (33)	–	–	–	Cardiomegaly, lung, liver and kidney congestion, pulmonary edema with hemorrhagic extravasation, multi-organ congestion	Concentric intimal hyperplasia, generalized multi-organ congestion		No specific findings
Lichtenfeld 2016 (34)	–	–	–	Cardiomegaly, epicardial fat, marked ventricular hypertrophy, multifocal endocardial fibrosis in atria	Enlarged myofibers, pleomorphic nuclei, expanded interstitial tissue, increased capillary network		No specific findings
Hernandez-Guerra et al. 2019 (7)	15	5	15	Cardiomegaly, severe coronary atherosclerosis, occlusive thrombosis, myocardial infarction	Myocyte hypertrophy, intramyocardial vessel disease, scarring, acute and subacute myocardial infarction		No specific findings
Lusetti et al. 2015 (35)	20	5	16	Prominent muscular masses, normal coronary anatomy, no acute myocardial infarction	Interstitial fibrosis, perivascular fibrosis, fibroadipose metaplasia		Occasional single myocyte necrosis
Lusetti et al. 2018 (36)	–	–	–	Prominent muscular masses, LV hypertrophy, multifocal endocardial fibrosis in atria	Myocyte necrosis, interstitial fibrosis		No specific findings
Lusetti et al. 2018 (36)	–	–	–	Prominent muscular masses, coronary artery fatty streaks	Intima and media thickening		No specific findings
Lusetti et al. 2018 (36)	–	–	–	Prominent muscular masses, LV hypertrophy	Necrosis of group of myocytes		No specific findings
Lusetti et al. 2018 (36)	–	–	–	Prominent muscular masses, LV and RV hypertrophy, myocardial fibrosis	Myocyte necrosis, interstitial fibrosis		No specific findings
Lusetti et al. 2018 (36)	–	–	–	Prominent muscular masses, testicular atrophy	No myocardial infarction observed		No specific findings
Ferenchick et al. 1991 (37) (by Frati et al. (1)	16	4	–	Marked cardiac hypertrophy, myocardial fibrosis	Contraction band necrosis, lymphocytic infiltration		Not specified
Luke et al. 1990 (38) (by Frati et al. (1)	–	–	–	Occlusive thrombus in left coronary artery	Not specified		Not specified
Kennedy et al. 1993 (39) (by Frati et al. (1)	13	4	17	Hypertrophic cardiomyopathy	Myocarditis, focal fibrosis		Not specified
Kennedy et al. 1993 (39) (by Frati et al. (1)	17	6	12	Hypertrophic cardiomyopathy	Myocarditis, focal fibrosis		Not specified
Dickerman et al. 1996 (40) (by Frati et al. (1)	–	–	–	Left ventricular hypertrophy	Not specified		Not specified
Hausmann et al. 1998 (41) (by Frati et al. (1)	–	–	–	Right ventricle dilatation, focal endocardial induration	Disseminated focal necrosis, interstitial fibrosis, nuclear polymorphism		Not specified
Fineschi et al. 2001 (42) (by Frati et al. (1)	14	4	13	Myocardial infarction, hyperdistended myocardial cells	Contraction band necrosis, fibrotic microfoci		Not specified
Fineschi et al. 2001 (42) (by Frati et al. (1)	19	6	14	Hyperdistended myocardial cells, contraction band necrosis	Fibrotic microfoci in internal LV		Not specified
Di Paolo et al. 2007 (43) (by Frati et al. (1)	13	–	–	Severe interstitial and epicardial fibrosis, myocardial bridge	Hypertrophy in non-fibrotic areas		Not specified
Di Paolo et al. 2007 (43) (by Frati et al. (1)	11	–	–	Focal interstitial fibrosis, mild coronary intimal hyperplasia	Hypertrophic myocytes in fibrosis areas		Not specified
Di Paolo et al. 2007 (43) (by Frati et al. (1)	10	–	–	Unique granulation tissue focus, mild intimal hyperplasia in coronaries	Normal coronary arteries		Not specified
Di Paolo et al. 2007 (43) (by Frati et al. (1)	12	–	–	Moderate/severe epicardial fibrosis, focal interstitial fibrosis	Myocytes hypertrophy, occasional basophilic degeneration		Not specified
Fineschi et al. 2007 (44) (by Frati et al. (1)	–	–	–	Contraction band necrosis, segmentation of myocardial cells	Subendocardial LV fibrosis, widening of intercalated discs		Not specified
Fineschi et al. 2007 (44) (by Frati et al. (1)	–	–		Contracted myocardial bundels, granular disruption of myocytes	Coronary scattered fatty streaks, focal myocardial fibrosis		Not specified
Thiblin et al. 2009 (45) (by Frati et al. (1)	–	–	–	Fatty thickening in proximal LAD, granulation tissue foci	Lymphocytic infiltration, intramural vessels		Not specified
Montisci et al. 2012 (46) (by Frati et al. (1)	15	4	16	Concentric LV hyperthrophy, patchy necrosis, lymphocitic infiltrates	Interstitial fibrosis, replacement fibrosis		Not specified
Montisci et al. 2012 (46) (by Frati et al. (1)	15	5	20	Biventricular eccentric hypertrophy, mild atrial dilatation	Colliquative myocytolisis		Not specified
Montisci et al. 2012 (46) (by Frati et al. (1)	16	5	18	Biventricular dilatation, bizarre myocyte nuclei	Fibrofatty replacement		Not specified
Montisci et al. 2012 (46) (by Frati et al. (1)	13	3.5	16	Multiorgan congestion, LAD stenosis 50%	Polymorphous inflammatory infiltrates		Not specified

RW, right ventricle; LV, left ventricle; IS, interventricular septum; WT, wall thickness.

Analyzing Table 4, a total of 35 cases were identified. The age of the subjects whose death was associated with AAS abuse ranged from 13 to 39 years, and they were predominantly male, with the exception of those described in Case 11 and Case 15 of the review by Frati et al. (1). When examining the BMI data, where available, values ranged from 25 to 41, indicating that the individuals involved were overweight or obese. However, because the body mass index classifies individuals only quantitatively rather than qualitatively, it does not provide information on actual body composition; it therefore cannot distinguish whether a high BMI reflects greater fat mass or increased muscle mass.

Under normal conditions, the heart weighs between 280 and 340 g in adult males and between 230 and 280 g in adult females. With regard to autopsy findings, in many deceased subjects, the heart weight exceeded these limits; in several cases, it ranged from 400 to 500 g, and in one particular case, it reached 900 g (1, 46), indicative of hypertrophy/cardiomegaly.

Other notable cardiac findings included ventricular hypertrophy. Concentric or eccentric left ventricular hypertrophy was noted frequently, although some studies also reported right ventricular hypertrophy or even biventricular dilation. As for the coronary arteries, many cases demonstrated normal vessels; however, some studies highlighted atherosclerotic changes (e.g., severe coronary atherosclerosis, intimal hyperplasia, occlusive thrombosis, etc.). Endocardial fibrosis—particularly in the atria—was also observed in certain instances. In other organs, the livers of many deceased individuals were congested and steatotic, while pulmonary congestion, edema, or emphysema were reported in other cases. Renal congestion or hemorrhage was occasionally documented. As well testicular atrophy was found (35, 36). Multiorgan congestion was especially evident in the presence of acute or subacute heart failure.

From a histological standpoint, a substantial array of myocardial abnormalities was recorded. One of the most commonly observed features was cardiomyocyte hypertrophy, characterized by enlarged myocytes with pleomorphic nuclei; in some instances, the myocytes exhibited a “hyperdistended” or “bizarre” morphology. As reported in the study by Basso and colleagues, cardiomyocyte hypertrophy is characterized by a cell diameter—measured in cross-section at the nuclear level—exceeding 15 µm. Additionally, the nuclei appear enlarged and hyperchromatic due to increased DNA ploidy resulting from enhanced DNA replication without subsequent mitosis (47). Fibrosis was likewise reported: interstitial fibrosis was most common, but perivascular, subendocardial, epicardial, and replacement fibrosis (i.e., fibrous scarring replacing necrotic myocytes) were also identified. Among the necrotic/scarring phenomena, contraction band necrosis was frequently noted—often linked to stress, elevated catecholamine levels, or ischemia-reperfusion injury—and, in a few cases, “granular disruption of myocytes” or “basophilic degeneration” were reported. Inflammatory infiltrates were also detected, predominantly lymphocytic but also macrophagic [particularly according to Cecchi et al. (28)]; in some instances, they were described as “polymorphic.” Finally, mention should be made of vascular changes, including intimal hyperplasia, medial thickening, and the presence of “fatty streaks” in coronary arteries (which is especially unusual in young individuals), as well as small intramural vessel disease.

Immunohistochemical analyses are not reported in the majority of the examined studies; however, the few that include these techniques [e.g., Cecchi et al. (26)] document the presence of CD68-positive macrophages, TUNEL-positive cells, and Caspase-3 activation. The detection of CD68-positive macrophages indicates an inflammatory or necrotic process, while TUNEL positivity reflects DNA fragmentation associated with apoptosis. In addition, Caspase-3 immunoreactivity further corroborates the activation of apoptotic pathways in some cardiomyocytes. Overall, these findings suggest that ongoing myocyte death (via apoptosis and/or necrosis) and inflammatory events are likely amplified by anabolic steroid use.

To sum up, the evidence indicates that cardiac remodeling is a predominant feature among anabolic steroid users, as demonstrated by significant increases in heart weight and wall thickness—particularly affecting the left ventricle—pointing to pathological hypertrophy. Frequent interstitial, perivascular, and replacement fibrosis reflects the chronic nature of myocardial damage. Inflammatory and necrotic alterations are also evident; indeed, lymphocytic infiltration, contraction band necrosis, and inflammatory-mediated myocyte injury have been documented, suggesting processes that resemble myocarditis or toxic/inflammatory injury. Although not consistently observed in every case, premature atherosclerosis or intimal thickening of the coronary arteries emerges as a concern among younger individuals abusing anabolic steroids, sometimes culminating in myocardial infarction or occlusive coronary events at unexpectedly early ages. Additional organ pathology includes hepatic and renal congestion, as well as testicular atrophy, underscoring the systemic consequences of prolonged anabolic steroid exposure. From a mechanistic perspective, histological findings (e.g., fibrosis and necrosis) and immunohistochemical markers of apoptosis suggest that anabolic steroids can exert direct toxic effects on cardiomyocytes, contribute to microvascular alterations within the coronary circulation, and foster pro-inflammatory or pro-thrombotic states, ultimately exacerbating cardiac dysfunction.

In the Table 4 the collective findings from these reports underscore the severe cardiovascular and systemic complications associated with anabolic-androgenic steroid (AAS) misuse, particularly among young adults. Pathological examination repeatedly demonstrated concentric and, in some instances, dilated cardiomyopathy, with frequent detection of myocyte hypertrophy, myocardial fibrosis, and evidence of ongoing myocardial injury such as myocytolysis. Immunohistochemical analysis, primarily utilizing antibodies against troponin T, revealed inflammatory and fibrotic changes that contribute to arrhythmias and sudden cardiac events. Toxicological assessments predominantly detected various AAS compounds, often in conjunction with additional substances such as ephedrine, clenbuterol, tamoxifen, or even 2,4-dinitrophenol, suggesting the possibility of synergistic harmful effects on the cardiovascular system. In many cases, coronary atherosclerosis, thrombosis, and conduction anomalies were found at autopsy, reinforcing the notion that AAS use precipitates structural and functional derangements of the heart. Collectively, these observations support the hypothesis that chronic AAS administration exerts direct cardiotoxic effects, promotes pathological remodeling, and predisposes abusers to fatal cardiac arrhythmias and ischemic events.

**Figure 3:**
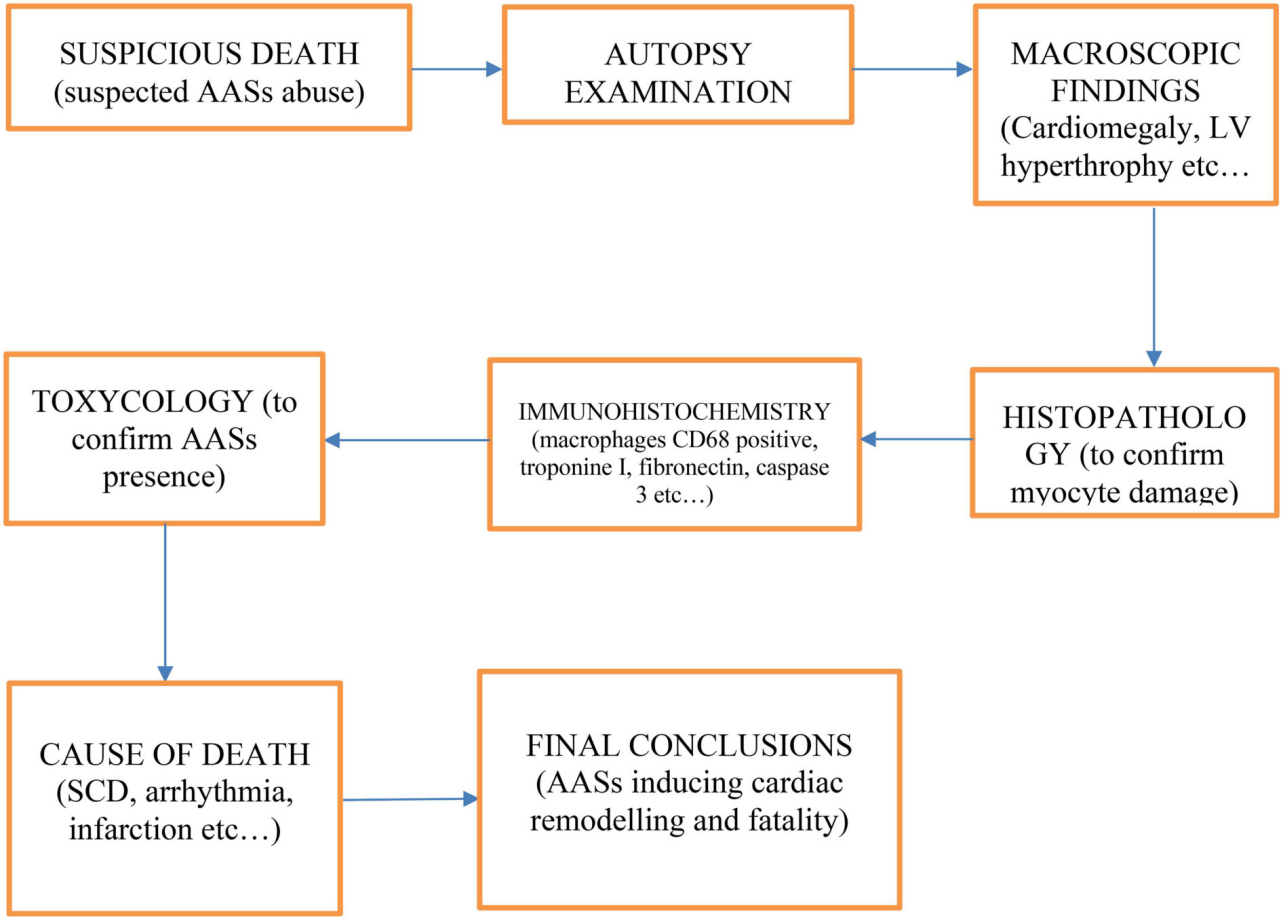
Diagnostic flowchart to investigating deaths in which anabolic-androgenic steroid (AAS) abuse is suspected.

Thanks to the information contained in the articles cited in this review, the following diagnostic flowchart was created (Figure 3), taking into account anamnestic data, autopsy findings (both macroscopic and microscopic), as well as histological, immunohistochemical, and toxicological elements:

1. Suspicious death;
2. Autopsy examination;
3. Macroscopic findings (e.g.: Cardiomegaly, LV hypertrophy, coronary thrombosis);
4. Microscopic findings (e.g.: Fibrosis, disarray);
5. Histopathology (to confirm myocyte damage);
6. Immunohistochemistry (e.g.: macrophages CD68 positive, Troponin I, Fibronectin, Caspase 3);
7. Toxicologic analysis (to confirm AASs presence);
8. Cause of death determination (e.g.: SCD, arrhythmia, Infarction);
9. Final conclusions (AASs inducing cardiac remodeling and fatality).

This diagnostic flowchart outlines a systematic approach to investigating deaths in which anabolic-androgenic steroid (AAS) abuse is suspected. Following the initial identification of a suspicious death, autopsy examination provides both macroscopic and microscopic evidence—ranging from cardiomegaly and left ventricular hypertrophy to fibrosis and myocyte disarray—that may suggest steroid-related cardiac remodeling. Histopathological and immunohistochemical assessments (e.g., for macrophages CD68, Troponin I, Fibronectin, Caspase 3) further define the extent and nature of tissue damage, enabling the differentiation of AAS-induced effects from other etiologies. Toxicological analysis is essential to confirm the presence of AASs. Ultimately, these findings converge to determine the cause of death—whether sudden cardiac death, arrhythmia, infarction, or other pathology—and to draw final conclusions regarding the role of AASs in cardiac remodeling and fatal outcomes.

## 5 Conclusions

**Figure 4:**
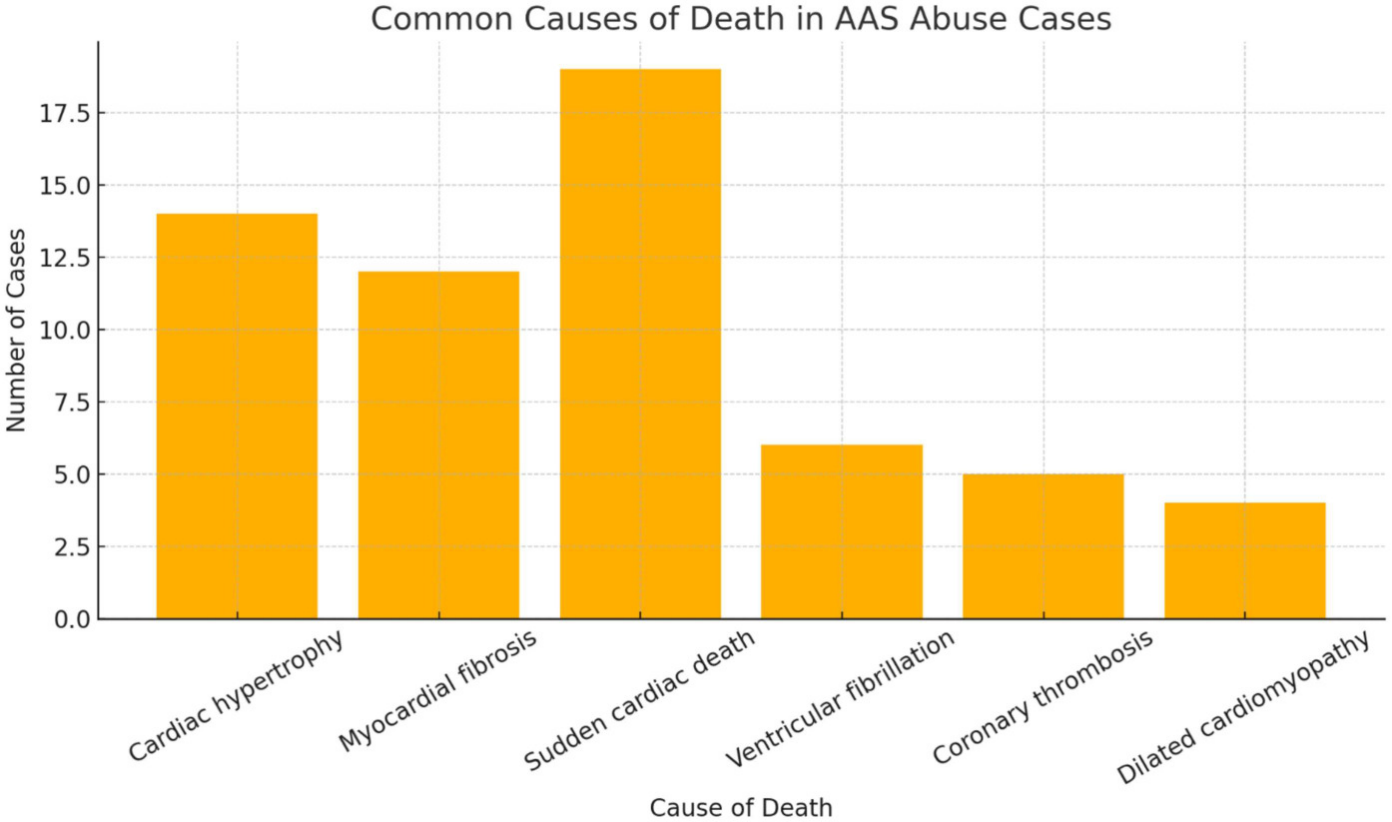
Common causes of death in AAS abuse cases worldwide.

Studies have consistently highlighted the severe cardiovascular risks associated with AAS abuse. Across different study populations (including bodybuilders, athletes, and adolescents), autopsy findings have revealed recurring patterns of left ventricular hypertrophy, myocardial fibrosis, and cardiomegaly, often in the absence of significant coronary pathology. These structural changes predispose AAS users to the development of cardiac arrhythmias, myocardial infarctions, and sudden cardiac death (SCD), often triggered by ventricular fibrillation or coronary thrombosis (Figure 4).

Histopathological analyses have further reinforced the connection between chronic AAS use and cardiomyocyte necrosis, myofibrillar disarray, and interstitial fibrosis, indicative of long-standing myocardial remodeling. In some cases, immunohistochemical studies have demonstrated the presence of fibronectin, troponin, and complement markers (C5b-9), suggesting that subclinical myocardial damage evolves over time.

Post-mortem toxicological investigations have confirmed the presence of multiple AAS compounds (nandrolone, testosterone, stanozolol, and trenbolone) in biological samples, often accompanied by polypharmacy, including psychoactive substances and recreational drugs.

This combination exacerbates the likelihood of fatal cardiovascular events, underscoring the complex interplay between AAS abuse and other pharmacological agents.

Despite the absence of acute ischemic events in many cases, the findings suggest that AAS-induced hypertrophy and fibrosis independently contribute to the pathogenesis of fatal arrhythmias. The lack of pre-existing cardiovascular conditions in many AAS users emphasizes the direct role of these substances in promoting myocardial damage.

This review underscores the critical need for heightened awareness among clinicians, forensic pathologists, and public health officials regarding the potentially fatal consequences of AAS abuse. The integration of autopsy, histopathology, immunohistochemistry, and toxicology in forensic investigations remains essential for accurately characterizing AAS-related fatalities and guiding prevention strategies.

Alongside the diagnostic elements analyzed in this article, many others have been already developed. For example, PMMRI (Post-Mortem Magnetic Resonance Imaging) has shown promising results in the study of the myocardium in SCD (48). Also, PMCMR (Post-Mortem Cardiac Magnetic Resonance) has given good results (49). In any case of sudden cardiac death, a panel of genetic tests should always be considered to exclude underlying hereditary conditions (50).

Further research is necessary to elucidate the molecular mechanism underlying AAS-induced cardiac remodeling, as well as to establish preventive measures and intervention strategies aimed at reducing AAS misuse, particularly among young athletes and bodybuilders.
